# Plasma Exosome Gene Signature Differentiates Colon Cancer from Healthy Controls

**DOI:** 10.1245/s10434-023-13219-7

**Published:** 2023-03-02

**Authors:** Paul A. Vallejos, Amber Gonda, Jingjing Yu, Brittany G. Sullivan, Arsha Ostowari, Mei Li Kwong, Audrey Choi, Matthew J. Selleck, Janviere Kabagwira, Ryan N. Fuller, Daniel J. Gironda, Edward A. Levine, Christopher C. W. Hughes, Nathan R. Wall, Lance D. Miller, Maheswari Senthil

**Affiliations:** 1 Department of Basic Science, Division of Biochemistry, Loma Linda University School of Medicine, Loma Linda, CA USA; 2 Center for Health Disparities and Molecular Medicine, Loma Linda University School of Medicine, Loma Linda, CA USA; 3 Department of Surgery, Division of Surgical Oncology, University of California, Irvine Medical Center, Orange, CA USA; 4 Division of Surgical Oncology, Loma Linda University Health, Loma Linda, CA USA; 5 Department of Cancer Biology, Wake Forest Health, Winston-Salem, NC USA; 6 Department of Surgery, Division of Surgical Oncology, Wake Forest Health, Winston-Salem, NC USA; 7 Department of Molecular Biology and Biochemistry, University of California, Irvine, Irvine, CA USA

## Abstract

**Background:**

Liquid biopsies have become an integral part of cancer management as minimally invasive options to detect molecular and genetic changes. However, current options show poor sensitivity in peritoneal carcinomatosis (PC). Novel exosome-based liquid biopsies may provide critical information on these challenging tumors. In this initial feasibility analysis, we identified an exosome gene signature of 445 genes (ExoSig445) from colon cancer patients, including those with PC, that is distinct from healthy controls.

**Methods:**

Plasma exosomes from 42 patients with metastatic and non-metastatic colon cancer and 10 healthy controls were isolated and verified. RNAseq analysis of exosomal RNA was performed and differentially expressed genes (DEGs) were identified by the DESeq2 algorithm. The ability of RNA transcripts to discriminate control and cancer cases was assessed by principal component analysis (PCA) and Bayesian compound covariate predictor classification. An exosomal gene signature was compared with tumor expression profiles of The Cancer Genome Atlas.

**Results:**

Unsupervised PCA using exosomal genes with greatest expression variance showed stark separation between controls and patient samples. Using separate training and test sets, gene classifiers were constructed capable of discriminating control and patient samples with 100% accuracy. Using a stringent statistical threshold, 445 DEGs fully delineated control from cancer samples. Furthermore, 58 of these exosomal DEGs were found to be overexpressed in colon tumors.

**Conclusions:**

Plasma exosomal RNAs can robustly discriminate colon cancer patients, including patients with PC, from healthy controls. ExoSig445 can potentially be developed as a highly sensitive liquid biopsy test in colon cancer.

**Supplementary Information:**

The online version contains supplementary material available at 10.1245/s10434-023-13219-7.

Cancer is a leading cause of death worldwide and is the second leading cause of death in the United States (US). Colorectal cancer (CRC) is the second most common cause of cancer-related death in the US, with an estimated incidence of 147,500 new cases and 52,980 deaths in 2021.^1^ Although most patients with CRC have excellent survival when diagnosed at early stages and undergo curative resection, nearly 25% of patients present with distant disease at diagnosis and another 35% develop recurrence after curative intent treatment.^2–4^

At present, liquid biopsies have become an integral part of cancer care as they offer a rapid and minimally invasive technique to detect and evaluate disease progression. First-generation liquid biopsies utilizing cell-free circulating tumor DNA (ctDNA) have shown promise in identification of molecular-residual disease (MRD), somatic gene alterations, and to assess treatment response in CRC; however, there are significant limitations. Specifically, ctDNA has poor sensitivity in mucinous cancers^5^ and peritoneal carcinomatosis (PC),^6^ with ctDNA detected in only 38–53% of patients with PC.^7, 8^ We recently reported that ctDNA is detected at significantly lower levels in PC compared with visceral metastasis (VM) and is unreliable to detect somatic gene alterations in gastrointestinal PC.^6^ Due to its dismal prognosis and the only site of disease in 44% of patients with CRC recurrence,^9^ there is an urgent need to identify novel blood-based biomarkers that can accurately identify colon cancer regardless of tumor type and metastasis location.

Exosomes have gained popularity in recent years as potential circulating biomarkers in cancer. Exosomes are stable lipid-bilayer bound nanovesicles (30–150 nm), released by most cells and found in blood, urine, and other bodily fluids.^10^ Exosomes are released more abundantly by cancer cells and have been reported to carry important information for cancer cell–cell communication and progression.^11–13^ Exosome contents are rich in nucleic acids, proteins, and lipids, and have similarities to the donor cell, and hence show great promise as a liquid biopsy tool for the evaluation of labile biomarkers (protein and RNA).

We hypothesize that plasma exosome gene expression in patients with colon cancer will allow us to accurately detect the presence of disease, including PC. In this study, we sought to identify unique transcriptome profiles in peripheral plasma exosomes of patients with colon cancer grouped into non-metastatic (NM), metastatic to liver/lung (VM), and metastatic to the peritoneum (PC) compared with healthy controls.

## Methods and Materials

### Patient Samples

Patients with colon cancer were divided into those with localized disease (NM, *n* = 14), metastases to liver and lung (VM, *n* = 16), and PC (*n* = 12). Healthy volunteers who have no personal or family history of CRC or inflammatory bowel disease were included as controls (*n* = 10). Healthy volunteers were included to assess the relative differential expression of genes in patients. Frozen plasma samples collected immediately prior to primary resection, metastasectomy or cytoreduction for the NM, VM, and PC groups, respectively, were obtained. All samples were obtained under the auspices of Institutional Review Board (IRB)-approved studies, following the documentation of informed consent in accordance with institutional policies.

### Exosome Isolation and Characterization

Exosomes were isolated from patient plasma using ExoQuick (Systems Biosciences [SBI], Palo Alto, CA, USA; cat#EXOQ20A-1) following the manufacturer’s protocol, as previously reported.^14–16^ Briefly, plasma samples were thawed on ice and treated with thrombin for defibrination. The serum-like samples were then centrifuged at 1500 × g at 4°C to remove any leftover debris. Cleared serum was treated with ExoQuick overnight at the recommended ratio. The sample was then centrifuged at 1500 × g for 30 min to pellet the exosomes. The pellet was then resuspended in 250 µL of 0.22 µm filtered phosphate buffered saline (PBS) for further analysis.

### Nanoparticle Tracking Analysis

Validation of exosome isolation was performed using the NanoSight NS300 (Malvern, Inc., Malvern, UK) for size and concentration as previously reported,^17^ with instrument settings as follows: software NanoSight NTA 3.2, camera level 13–15, detection threshold 5, capture time 60 s, captures 5, flow rate 30. Vesicles were diluted at a ratio of 1:1000 in filtered PBS to achieve optimal concentrations of uniform particle distribution for accurate sizing analysis. The sample was sonicated for 30 s in a sonicating water bath and analyzed. Significance was tested with a one-way analysis of variance (ANOVA) followed by a post hoc Tukey test, as well as a two-tailed Student *t*-test with unequal variance, using a *p* value of <0.05.

### Western Blot Analysis

For Western blots of purified exosomes, vesicles were solubilized with lysates loaded onto gels according to protein concentration and nanoparticle tracking analysis (NTA) quantification. For protein concentrations, the BCA assay (Pierce, Rockford, IL, USA; 23225) was used as previously described.^15^ Proteins (20–40 µg) were separated using 12% Bis–Tris polyacrylamide gels, transferred onto polyvinylidene difluoride membranes (Millipore, Danvers, MA, USA) and probed using the following antibodies: rabbit monoclonal anti-CD63 (1:1000, SBI; EXOAB-CD63A-1), mouse monoclonal anti-Calnexin (1:1000, Santa Cruz Biotechnology, Dallas, TX, USA; sc-23954). Secondary antibodies (IR-Dye conjugated) were goat anti-mouse and goat anti-rabbit immunoglobulin (LICOR, Lincoln, NE, USA). Immunoreactive bands were detected using the Odyssey imaging system (LICOR) and quantified using ImageQuant software.

### Transmission Electron Microscopy

Purified exosome preparations (10 µL) were fixed with an equal volume of 4% paraformaldehyde at 4 °C for 30 min then diluted in a ratio of 1:3 with ddH_2_O. The fixed exosomes were then carefully placed on a carbon-coated 200-mesh copper grid for 20 min. The grids were contrasted with 1% uranyl acetate and then washed. The morphology of isolated exosomes was visualized with transmission electron microscopy (TEM; Talos L120C, Thermo Scientific, Waltham, MA, USA). The images of exosomes obtained from TEM were analyzed by ImageJ software to calculate the radius of exosomes.

### Next-Generation Sequencing (NGS) and Bioinformatics Analysis

Exosomes were processed for total RNA isolation using the SeraMir Exosome RNA Purification Column kit (SBI; RA806TC-1) according to the manufacturer’s instructions. RNA sequencing (RNA-seq) of 52 exosomal plasma samples was performed using paired-end sequencing on an Illumina HiSeq (Illumina, Inc., San Diego, CA, USA). Library preparation and RNA-seq was performed by SBI’s next-generation sequencing (NGS) services (SBI). Reads were aligned using BowTie2^18^ to GRCh37/hg19. Read count normalization (by median of ratios) and differential expression analysis were performed using the R/Bioconductor^19^ software package, DESeq2.^20^ Principal component analysis (PCA) was conducted using ClustVis software.^21^ The Bayesian Compound Covariate Predictor (BCCP)^22^ was used as implemented in the BRB-ArrayTools package (v4.6.0) developed by Dr. Richard Simon (National Cancer Institute [NCI]) and the BRB-ArrayTools Development Team.^23^ The ExoSig445 gene group was identified as genes overexpressed in cancer cases determined by DESeq2 as having baseMean read values > 10, and with Benjamini–Hochberg adjusted *p* values < 0.0001 for any one pairwise comparison of the controls versus a cancer type (NM, PC, or VM).

### The Gancer Genome Atlas Colon Adenocarcinoma Gene Expression Comparison

The NCI Genomic Data Commons (GDC) project—The Cancer Genome Atlas Colon Adenocarcinoma (TCGA-COAD)—was accessed using the GDC harmonized RNAseq data.^24^ TCGA consortium mapped to 57,000 transcripts for the COAD dataset, as well as each of the other 19 comparison sets (electronic supplementary material [ESM] Table 1). Gene-to-gene mapping was performed between ExoSig445 and TCGA-COAD. Initial comparison of common gene symbols identified 130 genes. Using the HUGO Gene Nomenclature Committee (HGNC) multi-symbol checker,^25^ alternate symbols were identified and 9 additional genes were found, resulting in a total of 139 common genes. Of these 139 genes expressed in both datasets, 58 were found to be overexpressed in colon cancer tissue in comparison with normal adjacent tissue in TCGA database. Several non-standard gene symbols in the ExoSig445 signature, such as DIAPH3-AS1:antisense or LOC100129636:copy41, were excluded from the gene-to-gene mapping. In preparation for comparison of the COAD database with those of other cancer types, redundant samples were removed from each dataset. The Student *t*-test and one-way ANOVA with post hoc Tukey were used to evaluate gene expression levels between cancer types and with gene expression between cancer and controls. Significance was established with a *p*-value of <0.05.

## Results

### Patient and Tumor Characteristics

Of the 42 patients with colon cancer, 14 had localized disease (NM), 16 had VM, and 12 had PC. The median age of patients in the NM, VM, and PC groups was 67, 58, and 56 years, respectively. The median age of the healthy controls (*n* = 10) was 30. In our cohort, including healthy controls, 55.8% were male and 51.9% were non-Hispanic White (Table 1).

**Table 1:** Study participant characteristics

	Healthy controls [*n* = 10]	NM [*n *= 14]	VM [*n *= 16]	PC [*n *= 12]
*Age, years*				
Median	30	67	58	56
Range	(20–50)	(45–82)	(41–69)	(32–63)
*Sex*				
Male	5	5	12	7
Female	5	9	4	5
*Ethnicity*				
Non-Hispanic White	5	5	9	8
Non-Hispanic Black	2	1	1	1
Asian/Pacific Islander	2	5	3	0
Hispanic	1	3	3	3
*Histology*				
Low-grade/well-differentiated	–	7	3	4
Moderately differentiated	–	5	13	4
High grade/poorly differentiated	–	2	–	4
*Sidedness*				
Right	–	10	10	6
Left	–	4	5	5
Both	–	–	1	1
*KRAS*				
Wild-type	–	3	7	4
Mutant	–	–	7	8
Unknown	–	11	2	–
*BRAF*				
Wild-type	–	–	8	6
Mutant	–	–	–	–
Unknown	–	14	8	6
*Chemotherapy*				
No	–	13	1	1
Yes	–	1	15	11

*NM* non-metastatic, *VM* visceral metastasis, *PC* peritoneal carcinomatosis

Among the patients with colon cancer, 61.9% had right-sided disease, 33.3% had left-sided disease, and 4.8% had disease on both sides. Moderately or poorly differentiated tumors were 66.7% of the total, and 35.7% had a KRAS mutation. The majority of colon cancer patients with metastasis included in this study had also undergone chemotherapy treatment (93%) (Table 1).

### Isolation Procedure Generates High Purity Populations of Exosomes

**Fig. 1:**
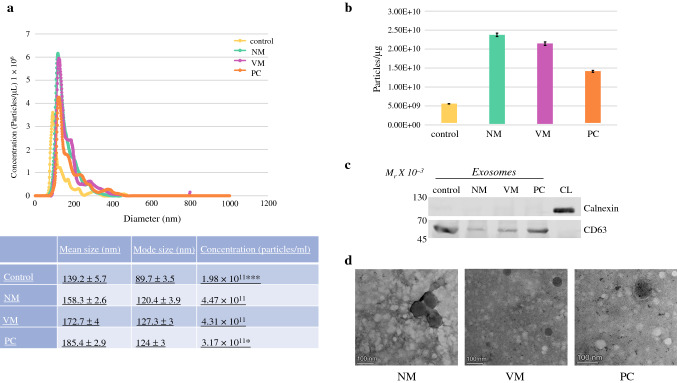
Characterization of extracellular vesicles from presurgical plasma samples from colon cancer patient and healthy volunteer samples. (**a**) Histogram and table of quantitative analysis of vesicle diameter and concentration in particles per milliliter from nanoparticle tracking analysis. Control vesicle concentration is extremely significantly less (*p* = 0.0000137), as determined by one-way ANOVA and a post hoc Tukey test, than the tumor-bearing samples. PC is also significantly less than VM (*p* = 0.027) and NM (*p* =0.014). (**b**) Exosome purity ratio was calculated as particles per microgram of protein ± standard error of the mean. (**c**) Proteins from vesicles were analyzed by immunoblotting using exosomal marker CD63 (positive control) and endoplasmic reticulum marker Calnexin (negative control), as well as CL. (**d**) Negative stain transmission electron microscopy of colon cancer patient exosomes. *NM* non-metastatic, *VM* visceral metastases, *PC* peritoneal carcinomatosis, *CL* cell lysate, *ANOVA* analysis of variance

Isolated exosomes were evaluated for size using NTA with a NanoSight NS300. Small volumes of four samples from each group were pooled for the exosome characterization analysis. Exosome populations and concentrations (Fig. 1a) were compatible with that previously reported for exosomes by our laboratory and others.^14, 16, 26^ Indicative of the main population in the isolate, the mode vesicle diameter for each group was as follows: healthy control, 89.7 nm (± 3.5 nm); NM, 120.4 nm (± 3.9 nm); VM, 127.3 nm (± 3 nm); and PC, 124 nm (± 3 nm). Finally, the exosome concentration measured on the NanoSight showed that the healthy control group (1.98 × 10^11^ particles/mL) had extremely significantly lower amounts of vesicles than all three of the tumor-bearing groups (ANOVA *p* = 0.0000137). Interestingly, the PC exosome concentration (3.17 × 10^11^ particles/mL) was significantly lower than both the NM (4.47 × 10^11^ particles/mL, *p* = 0.014) and VM groups (4.31 × 10^11^ particles/mL,* p* = 0.027)

To assess EV purity, a BCA assay was performed following isolation. The exosome purity ratio was calculated to be as follows: healthy control, 5.56 × 10^9^ particles/ug; NM, 2.37 × 10^10^ particles/ug; VM, 2.14 × 10^10^ particles/ug; and PC 1.41 × 10^10^ particles/ug (Fig. 1b). This is in line with the literature for the purity of EV capture using ExoQuick.^27^ To avoid possible contamination of our fraction by subcellular components, which are typically from the mitochondria, Golgi, or apoptotic bodies, we evaluated the EVs/exosomes for a known exosomal protein marker (CD63) and one endoplasmic reticulum marker (Calnexin) (Fig. 1c). Our results indicate that the vesicle preparation was enriched for those proteins known to be exosomal, with no ER contamination. To further characterize the isolated vesicles, TEM was performed to verify morphology and size (Fig. 1d). Taken together with the isolation technique, size, protein expression, and morphology, our results indicate that these vesicles can be considered exosomes.

### Differential Expression Analysis of Total RNA

**Fig. 2:**
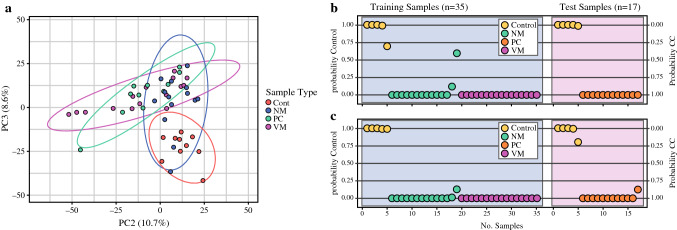
Classification of CC exosomal subtypes. (**a**) Delineation by unsupervised PCA. Genes were selected based on baseMean read values >100 (*n* = 14,898) and ranked by standard deviation across all samples. PCA was performed using 3000 exosomal genes with greatest expression variance across samples (i.e., standard deviation + 0.812–3.314). (**b, c**) Class prediction using a Bayesian probabilistic classifier of control and colon cases. To examine the potential reproducibility of a classification model, a training set comprising of healthy control, NM and VM cases was used to construct classification models by leave-one-out cross-validation to distinguish control from colon cases. Model performance was evaluated using a separate testing set containing healthy control and PC samples. Two models, differing by the significance threshold used for gene selection, were evaluated in parallel. (**b**) A significance threshold of *p* < 0.0001 (*n* = 198 genes) was used to construct the classifier. (**c**) A significance threshold of *p* < 0.00001 (*n* = 78 genes) was used to construct the classifier. Posterior probabilities computed for class assignment are shown for each sample. Both models predicted PC with 100% accuracy compared with healthy controls. *Cont* control (*n* = 10), *NM* non-metastatic (*n* = 14), *VM* visceral metastasis (*n* = 16), *PC* peritoneal carcinomatosis (*n* = 12). *CC* colon cancer, *PCA* principal components analysis

The RNA cargo of isolated exosomes was profiled by RNA-seq. Unsupervised principal components analysis (PCA) was used to investigate transcriptomic similarities and differences among patient and control samples in an unbiased fashion. For this purpose, PCA was performed using 3000 genes that exhibited the greatest expression variance across samples, regardless of sample origin. The PCA clustering revealed a striking separation between certain sample types. Healthy control samples clustered together, with some overlap with NM samples, but showed clear discernment from the PC and VM metastatic samples, the latter of which showed greater alignment with one another (Fig. 2a). Notably, this finding indicates that significant systemic variation in the RNA cargo of blood-derived exosomes exists between healthy individuals and cancer patients, and metastatic cancer patients in particular. Next, we employed a training and testing approach to construct classification models using a common classification algorithm based on the BCCP, and to validate model performance on randomly held-out samples. In this analysis, we created a training set that consisted of control, NM, and VM cases, and a separate test set consisting of control and PC cases. Leave-one-out cross-validation was used to create the classification models in the training set, and resulting models were evaluated in the test set. Two models, differing by significance threshold used for gene selection, were evaluated in parallel. In Fig. 2b, a significance threshold of *p* < 0.0001 (*n* = 198 genes) was used to construct the classifier, and in Fig. 2c, a significance threshold of *p* < 0.00001 (*n* = 78) genes was used to construct the classifier. Strikingly, both models resulted in the significant discernment of control and cancer patient samples, with a classification accuracy for control and PC samples of 100% (assuming a classification threshold of > 50% probability for class assignment).

**Fig. 3:**
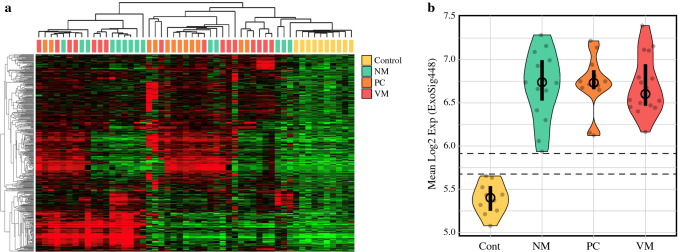
Evaluation of sample clustering by ExoSig445. (**a**) Cancer patient and healthy control samples (columns) were hierarchically clustered by average linkage analysis using exosomal RNAs (rows) identified as overexpressed in colon cancer cases as follows. DEGs were determined by DESeq2 analysis via pairwise group comparisons (i.e., Cont vs. NM, Cont. vs. PC and Cont vs. VM). Genes having baseMean read values >10, and with Benjamini–Hochberg adjusted *p*-values <0.0001 for any one pairwise comparison were identified. After filtering of prevalent and overlapping tRNA transcripts, 445 distinct transcripts (termed ExoSig445) were identified as highly significantly overexpressed in the colon cancer cases relative to controls. Multiple gene expression patterns were identified that clustered colon cancer samples into 5–6 distinct exosomal subtypes. Healthy control samples clustered apart from colon cancer samples due to a relative reduction in transcript levels. Red color indicates above mean expression; green color denotes below mean expression. Color intensity reflects the magnitude of expression level. (**b**) Violin plot of ExoSig445 expression scores defined as the per-sample arithmetic mean of the 445 genes. Black vertical bars indicate the interquartile range; black circle denotes the mean. Horizontal dashed lines indicate the range of no overlap in expression scores between healthy controls and colon cancer cases. *Cont* control (*n* = 10), *NM* non-metastatic (*n* = 14), *VM* visceral metastasis (*n* = 16), *PC* peritoneal carcinomatosis (*n* = 12), *DEGs* differentially expressed genes

Next, we applied a supervised approach to investigate differentially expressed genes (DEGs) between control and patient samples. We employed the DESeq2 algorithm and a pairwise comparisons strategy (i.e., control vs. NM, control vs. PC, and control vs. VM). The DEGs included mRNAs, microRNAs and lincRNAs with baseMean read values > 10 and false discovery rate (FDR)-adjusted *p* values < 0.0001. After filtering of highly prevalent and overlapping tRNA transcripts with strong internal correlation, 445 genes remained for downstream analyses and were termed ExoSig445 (Fig. 3a, ESM Table 2). As illustrated by supervised hierarchical clustering, the DEGs fully delineated the control samples from the cancer samples, primarily via reduced transcript levels in the control samples relative to the cancer samples. At a finer level of correlation, multiple gene expression patterns were observed to influence the further clustering of colon cancer samples into 5–6 semi-distinct exosomal subtypes. There were no significant differences in the gene expression between the KRAS mutant and wild-type groups. The BRAF status of the study population was either wild-type or unknown with a lack of patients with mutations to compare.

**Table 2:** Cancer-related functions of ExoSig445 and TCGA-COAD shared overexpressed genes

Tumor growth	Migration and invasion	Immune involvement	Metabolism	Cell death	Unknown
*BACE2^37^ *BMI1^38^ CCNC^39^ *DLGAP5^40^ EAF1^41^ HBS1L^42^ *LINC00210^43^ *MIR3182^44^ NAA40^45^ NHLRC3^46^ PTCSC3^47^ PTS^48^ *REXO4^49^ *SF3B1^50^ SIX6^51^ *SLC41A3^52^ *TAZ^53^ VAC14^54^ *WDR4^55^ ZNF691^56^	*ANGEL2^57^ *EIF4A1^58^ PKN3^59^ POMGNT1^60^ SETD6^61^ SHF^62^ SPAG1^63^ *STX6^64^	ADAM2^65^ C19orf66^66^ *CALCA^67^ *RBM12^68^ SNHG12^69^ TNFRSF9^70^	*ALDOB^71^ CDC25B^72^ MRPS14^73^ *PLA2G3^74^ RAET1K^75^ SELENOO^76^ SLC13A3^77^ SLC25A6^73^	*HSPBP1^78^ SLC41A1^79^ ZNF37BP^57^	AGPAT5 CLRN2 GPR89B KCVN2 MANSC1 METTL12 MFSD2B MIR6772 OCSTAMP SYNDIG1 TATDN2 UBFD1 ZSCAN25

^*^Multiple identified cancer functions

*TCGA-COAD* The cancer genome atlas colon adenocarcinoma

Evaluation of ExoSig445 expression scores (mean log2 expression of ExoSig445 genes) across colon cancer samples and healthy controls, as depicted in the violin plot (Fig. 3b), showed a clear delineation between the colon cancer groups and the healthy controls.

### Comparison Analysis of NGS Data with the Cancer Genome Atlas

Expression of ExoSig445 genes was compared with those found in TCGA-COAD harmonized cancer database. Using gene-to-gene mapping, 139 common genes were identified between the tumor tissue database and the plasma exosomal gene expression analysis. TCGA database provides gene expression analysis of normal adjacent tissue and upon comparison of the 139 common genes, 58 were overexpressed in colon cancer tissues, of which 53 were significantly overexpressed (*p* < 0.05, FDR-adjusted) (Table 2). The genes were grouped based on cancer-related function as reported in the literature (Table 2). Thirty of the overexpressed genes have been reported to have strong association with clinical outcomes in various cancers (ESM Table 3).

**Fig. 4:**
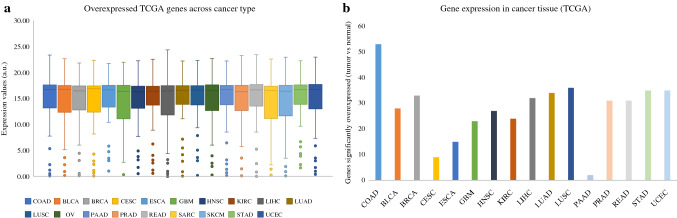
Comparison of ExoSig445 with TCGA. Of the 445 genes identified in ExoSig445, 139 were common to the TCGA-COAD database. Fifty-eight of those genes were overexpressed in tumor samples relative to adjacent non-malignant (normal) tissues. (**a**) When comparing the average expression values of the genes as a whole, no difference was found between COAD tumor expression and expression in other TCGA cancer types. (**b**) COAD tumor expression of individual genes was analyzed in comparison with normal tissue expression, and statistical significance was evaluated using Student’s *t*-test. Fifty-three of the 58 overexpressed genes were statistically significant in COAD. Other cancer types had a lower percentage of significant overexpression. OV, SARC, and SKCM did not have enough normal tissue in the database to conduct statistical analyses. *BLCA* bladder urothelial carcinoma, *BRCA* breast invasive carcinoma, *CESC* cervical squamous cell carcinoma and endocervical adenocarcinoma, *COAD* colon adenocarcinoma, *ESCA* esophageal carcinoma, *GBM* glioblastoma multiforme, *HNSC* head and neck squamous cell carcinoma, *KIRC* kidney renal clear cell carcinoma, *LIHC* liver hepatocellular carcinoma, *LUAD* lung adenocarcinoma, *OV* ovarian serous cystadenocarcinoma, *PAAD* pancreatic adenocarcinoma, *PRAD* prostate adenocarcinoma, *READ* rectum adenocarcinoma, *SARC* sarcoma, *SKCM* skin cutaneous melanoma, *STAD* stomach adenocarcinoma, *UCEC* uterine corpus endometrial carcinoma, *TCGA* The Cancer Genome Atlas

The 58 commonly overexpressed genes were then compared with expression levels in other cancer types using the same TCGA harmonized datasets. Similar expression levels were found among all 19 cancer types assessed without notable differences (Fig. 4a). However, individual gene analysis confirmed a higher percentage of genes (53/58, 91%) were overexpressed in the COAD dataset than in all the other cancer type datasets (Fig. 4b). Three of the 19 cancer types (ovarian cancer [OV], sarcoma [SARC], and skin cutaneous melanoma [SKCM]) did not have enough normal controls to facilitate the analysis and were hence omitted.

### Data Availability

The data generated in the development of ExoSig445 are available upon request. The data used for tissue comparison analysis were obtained from TCGA (https://portal.gdc.cancer.gov/).

## Discussion

In this study, we demonstrated that peripheral plasma exosomes from patients with colon cancer contain unique gene expression patterns that are distinctly different from healthy controls. We have also discovered that 58/445 overexpressed genes in exosomes (ExoSig445) were similarly overexpressed in colon tumor tissue, of which 30 genes have been reported to have significant association with clinical outcomes. Finally, we show that the shared overexpressed genes in exosomes from patients with colon cancer and colon tumor tissue are also shared among different cancer types, indicating common functional significance across cancers.

First-generation liquid biopsies offer easily accessible insight into tumor biology but are limited: cell-free DNA/ctDNA by stability and sensitivity, and circulating tumor cells by availability and heterogeneity.^28^ Release of ctDNA is considered mostly a passive process by shedding of DNA fragments from apoptotic or necrotic cells.^29^ On the other hand, exosomes are released by metabolically active cells,^30^ and are enriched with significant information that can provide a more accurate and inclusive understanding of tumor status.

We observed that the exosome concentrations were significantly higher in cancer groups compared with healthy controls, in concordance with other studies.^31^ The lack of concentration difference between the NM and VM groups is likely attributed to the fact that the majority of patients with metastatic disease (26/28, 93%) were treated with chemotherapy compared with patients in the NM group (1/14, 7%). However, it is interesting to note that the PC group had lower concentrations compared with the VM group despite similar chemotherapy conditions suggesting exosome concentration differences based on metastatic location. A recent prospective clinical study of plasma exosomes from patients with CRC showed that the exosome concentrations were higher in patients with metastatic disease compared with those with localized disease, and the concentrations decreased after chemotherapy. It was also noted that the exosome concentrations were influenced by the extent of metastatic disease and increased with disease progression.^32^

We observed that the exosomal gene expression patterns in metastatic disease were clearly different from the healthy controls. One of the key observations of this study is that the ExoSig445 gene signature found in colon cancer patient-derived exosomes can accurately detect the presence of disease, including in patients with PC, 100% of the time (Fig. 2). This is of major clinical significance as ctDNA has poor sensitivity in detecting PC,^7, 8^ and reliability in detection independent of tumor location is crucial for meaningful treatment decisions. To the best of our knowledge, this is the first study to evaluate plasma exosome gene expression in patients with colon cancer PC. These observations in combination with previous studies from our group in which we have shown the stability of exosomes across a wide range of sample collection conditions^33^ gives evidence of the utility of exosomes as a liquid biopsy tool.^34^

The comparison of ExoSig445 with colon tumor gene expression in TCGA database shows several important findings. Only a subset of genes overexpressed in the exosomes (58/445) was similarly overexpressed in the tumor tissue. This finding is expected, as exosomal loading is a highly regulated process resulting in a genetic profile that supports a function beyond simple replication of cellular/tissue character.^35, 36^ Therefore, comparison with tissue gene expression seeks not to find duplication but to identify validated biomarker genes within a novel exosomal gene signature. Nevertheless, the 58 overexpressed genes shared between exosomes and colon tumor tissue have important functions in cancer (Table 2). Further comparison conducted across common cancer types available in TCGA database demonstrated a remarkable consistency and uniformity of expression of the gene signature across multiple cancer types, suggesting a high correlation between gene expression and the processes of carcinogenesis. However, it is important to note that individual gene expression analysis demonstrated a higher percentage of these genes to be overexpressed in colon tumor tissue than the other cancer types, indicating signature specificity for colon cancer. Finally, these findings support the hypothesis that genes extracted from a heterogenous peripheral plasma exosomal population contain detectable tumor-specific biomarkers.

In summary, despite the inherent heterogeneity of plasma exosomes, we demonstrated that ExoSig445 is capable of differentiating patients with colon cancer, including PC, from healthy controls. Our study sheds light on the translational potential and the distinct advantages of exosome liquid biopsy. We are currently conducting a prospective, longitudinal clinical study in which patients with colon cancer are being monitored in various stages of treatment and surveillance to correlate exosome concentrations and gene signature with clinical outcomes. This will help refine the exosome gene signature and understand the predictive and prognostic value of the overall gene expression patterns. Further studies are also needed to evaluate the significance of the shared gene expression in plasma exosomes from patients with different cancers.

### Limitations

Since this is an initial exploratory study utilizing archived plasma samples, there are important limitations. The blood samples analyzed in this study were collected at a single time point (presurgical), therefore longitudinal changes in exosomal gene expression patterns could not be tested. The majority of patients with stage IV disease had received chemotherapy, and the gene expression patterns represent that of post-systemic treatment. The median age of healthy controls was younger than the median age of patients. We intentionally chose a younger cohort of controls to avoid the likelihood of undetected carcinogenesis that may occur with increasing age. Despite these limitations, our study has significant strengths. To the best of our knowledge, this is the first study to evaluate exosome gene signature expression levels in colon cancer and different metastatic states compared with healthy controls.

## Conclusion

We demonstrated that ExoSig445 can accurately separate patients with colon cancer from healthy controls. We have shown that the peripheral plasma exosome gene signature has the potential to be a liquid biopsy tool in colon cancer and may overcome the current challenges with sensitivity of ctDNA liquid biopsy in PC. Exosomal gene expression signatures may also provide valuable insights into tumor biology, treatment resistance, and prognosis. Further studies to refine this tool, along with validation in a larger cohort of patients, are currently underway.

## Supplementary Information

Below is the link to the electronic supplementary material.

Supplementary file1 (DOCX 63 KB)
