# Supplementary material for: Plasma Exosome Gene Signature Differentiates Colon Cancer from Healthy Controls
Source: Ann Surg Oncol. 2023 Mar 2;30(6):3833–44. doi: 10.1245/s10434-023-13219-7 (PMC10175396; doi:10.1245/s10434-023-13219-7)
Supplement: Supplementary file 1 — Supplementary file1 (DOCX 63 KB) [file 10434_2023_13219_MOESM1_ESM.docx]

**Supplemental table 1.** **TCGA cancer types and nomenclature** (<https://gdc.cancer.gov/resources-tcga-users/tcga-code-tables/tcga-study-abbreviations>)

| **BLCA** | Bladder Urothelial Carcinoma | **LUSC** | Lung squamous cell carcinoma |
| --- | --- | --- | --- |
| **BRCA** | Breast Invasive Carcinoma | **OV** | Ovarian serous cystadenocarcinoma |
| **CESC** | Cervical squamous cell carcinoma and endocervical adenocarcinoma | **PAAD** | Pancreatic adenocarcinoma |
| **COAD** | Colon adenocarcinoma | **PRAD** | Prostate adenocarcinoma |
| **ESCA** | Esophageal carcinoma | **READ** | Rectum adenocarcinoma |
| **GBM** | Glioblastoma multiforme | **SARC** | Sarcoma |
| **HNSC** | Head and Neck squamous cell carcinoma | **SKCM** | Skin cutaneous melanoma |
| **KIRC** | Kidney renal clear cell carcinoma | **STAD** | Stomach adenocarcinoma |
| **LIHC** | Liver hepatocellular carcinoma | **UCEC** | Uterine corpus endometrial carcinoma |
| **LUAD** | Lung adenocarcinoma |  |  |

**Supplemental table 2: Genes identified as ExoSig445**

| 5S_copy141 | DYNLL11 | linc-CHRM2-2 | LOC283194:antisense | RNF5:copy51 |
| --- | --- | --- | --- | --- |
| 5S_copy154 | EAF1 | linc-CHRNA7:antisense | LOC401177:antisense | RNF5:copy61 |
| 5S_copy265 | ECT2L1 | linc-CPEB2-10:copy2 | LOC728730:antisense | RNF5:copy71 |
| 5S_copy37 | EEF1E11 | linc-CPEB2-10:copy3 | LPXN | RNF51 |
| 5S_copy381 | EEF1G1 | linc-CPEB2-11 | LRRC3C | RNF5P1:antisense |
| 5S_copy42 | EFNA5 | linc-CPEB2-11:copy2 | LRRC43:copy2 | SCAI1 |
| ABCB101 | EIF3C:copy21 | linc-CSTB-7:antisense | LRRC9:antisense | SELO |
| ABI21 | EIF3E1 | linc-CTNNBL1-2:antisense | LRRCC11 | SEPTIN11 |
| ACA47 | EIF4A1 | linc-DNAH6-2 | LRRIQ4 | SEPTIN14 |
| ACBD71 | ENTPD41 | linc-DUSP10-1 | LRRTM1 | SEPTIN6 |
| ACOX31 | EVA1B1 | linc-EFEMP1-2:antisense | MAEA1 | SEPT6:copy2 |
| ADAM2 | FAHD11 | linc-ERGIC2:antisense | MANSC1 | SETD6 |
| AGPAT5 | FAM19A2 | linc-ETAA1-2:copy2 | MAPT1 | SETDB11 |
| AGTRAP1 | FAM214B1 | linc-EXD1:copy2:antisense | MARCHF11 | SF3B1 |
| AK91 | FAXC | linc-EXOC4-4:antisense | MARCHF4 | SFRP5 |
| AKAP3 | FMN21 | linc-FAM198B-1:antisense | MASP2:copy21 | SHF |
| AKAP7:copy21 | FNBP1L1 | linc-FAM19A5-8 | METTL12 | SHF1 |
| AKT31 | FOXE11 | linc-FANCL-6 | MFSD2B | SIMC1 |
| ALDOB | FOXI31 | linc-FBXO16-2:copy3 | MFSD6 | SIT1 |
| ALG14 | FRMD6-AS2:copy2 | linc-FBXO33-5:antisense | MIB21 | SIX6 |
| AMICA1 | GABRB11 | linc-FLI1-3 | MKLN1:copy21 | SLC13A3 |
| ANGEL2 | GBX11 | linc-FPGT-4:antisense | MKRN21 | SLC25A41 |
| ANKRD20A11P | GIF1 | linc-FRG1-12:antisense | MORC31 | SLC25A43 |
| ANKRD281 | GOPC | linc-GMPS-1:antisense | MRPS14 | SLC25A6 |
| APH1B1 | GPR89B | linc-GPR65-1 | MSRB11 | SLC25A6:copy2 |
| ASPH:copy2 | GPSM1:copy2 | linc-HNRNPA2B1-2:antisense | MTHFD2P1 | SLC26A10 |
| ATP5A1 | GPSM1:copy21 | linc-HOXD13-1:antisense | MUM1L1 | SLC41A1 |
| BACE2 | GSDMD1 | linc-IFT74-1 | MYF51 | SLC41A3 |
| BCAP29:antisense | GTF2B1 | linc-KCNV1-1:copy3:antisense | NAT11 | SLC52A1 |
| BEST41 | GUCY1A21 | linc-LAPTM4A-1:antisense | NBPF10 | SMPX |
| BHMT21 | GUSBP11 | linc-LHX2-1:antisense | NBPF101 | SNHG12 |
| BTD1 | GYG11 | linc-LRMP-1:antisense | NBPF24:copy2 | SPAG1 |
| C10orf1131 | GZMA | linc-MAGEB18 | NDST41 | SPPL3 |
| C11orf481 | HBS1L | linc-MAP1LC3B2-5:antisense | NEDD9:copy21 | SSC5D |
| C11orf73:antisense | HBS1L:copy2 | linc-MIA3-2:antisense | NELL2 | STMND11 |
| C17orf821 | HEPACAM21 | linc-NAP1L2-4:antisense | NFKB1 | STX6 |
| C19orf66 | HIST1H2APS1 | linc-NDST3-3:antisense | NHLRC3 | SYNDIG1 |
| C19orf811 | HLA-DPB1:copy2 | linc-NKAIN3-1:copy2:antisense | NOP161 | TAF5 |
| C2orf781 | HLA-DPB1:copy5 | linc-NLGN1:antisense | NPY5R1 | TATDN2 |
| C4orf451 | HLA-DPB1:copy7 | linc-NTSR2-1:antisense | NSA21 | TAZ |
| C7orf761 | HLA-DPB1:copy8 | linc-NUDT4-2 | OCSTAMP | THEMIS |
| C8orf311 | HPS11 | linc-NUSAP1:copy2 | OR8A11 | THUMPD31 |
| C9orf721 | hsa-mir-2277 | linc-OXCT1-1:copy3 | OSGIN21 | TIMD41 |
| CACNB2:copy4 | hsa-miR-3182 | linc-PARP4 | OTUD41 | TMCC2 |
| CACNG41 | hsa-miR-3654 | linc-PCDH10-1:antisense | PA2G4P4:antisense | TMEM1641 |
| CALCA | hsa-mir-6772 | linc-PER2-2:antisense | PADI2 | TMEM1741 |
| CASQ2 | hsa-miR-6772-3p | linc-PKN2-1 | PCBP41 | TMEM200A |
| CBLC | HSBP1L1 | linc-PPAPDC1A:copy4:antisense | PGD1 | TMEM254-AS1 |
| CCL3L3:copy21 | HSD17B4 | linc-PPIAL4A-4:antisense | PHEX1 | TMEM41A1 |
| CCL3L31 | HSP90B11 | linc-RALGAPA1-4 | piR-30113 | TMIE |
| CCL5 | HSPBAP11 | linc-RALGAPA1-4:copy2 | piR-43770 | TNFRSF9 |
| CCNC | HSPBP1 | linc-RALGPS2-2 | piR-61644 | TOP2B |
| CCNL2:copy2 | IL3RA | linc-RARRES3:antisense | PIWIL41 | TOR1AIP1 |
| CD1091 | IL3RA:copy2 | linc-RELN:antisense | PKN3 | TPTE2P3:antisense |
| CD1631 | INTS4L2 | linc-RGL4-1:antisense | PLA2G3 | TRIM9:copy21 |
| CD274 | JKAMP | linc-RGMB-2 | PLCH11 | TRIM91 |
| CD341 | KALRN | linc-RNF32-1:antisense | PLXND11 | TRPC5 |
| CDC25B | KCNV2 | linc-SATB2-2:copy2:antisense | PMS2L2:copy2 | U1:RF00003.104 |
| CEACAM21 | KDM1A1 | linc-SDCCAG1-2 | POMGNT1 | U50B |
| CLRN2 | KIAA0825 | linc-SLC22A11-2:antisense | PON3 | UBE2B |
| CLUL1 | KIAA0825:copy2 | linc-SLC25A26-1 | PPA11 | UBFD1 |
| CLUL1:copy2 | KLHL13 | linc-SLC25A26-1:copy2 | PPID | ULK21 |
| COL24A11 | KLHL241 | linc-SLC25A32-2 | PPM1K1 | UNC45B1 |
| COL25A1:antisense | LAIR11 | linc-SLC26A9-1:antisense | PPP1R12B | UPP2:copy21 |
| COL25A1:copy21 | LINC00174:antisense | linc-SLC7A11-4 | PPP1R12B:copy2 | USO11 |
| COLEC11 | LINC00210 | linc-SLITRK1-4:antisense | PPP1R3F1 | VAC14 |
| COLEC11:copy2 | LINC00324 | linc-SPO11-3 | PRICKLE2 | VAC141 |
| COLEC11:copy21 | LINC00568:antisense | linc-SPTLC2 | PRKCA | VCAM1 |
| COLEC11:copy31 | LINC00574:antisense | linc-SRBD1-2 | PTCSC3 | WDR4 |
| COLEC111 | LINC00598:antisense | linc-SUZ12-4 | PTGES3L1 | WDR661 |
| COLQ1 | LINC00869:antisense | linc-TMCC1-2:antisense | PTPLB | WT1-AS_4:RF02206.1 |
| CPNE61 | linc-ADM-1:antisense | linc-TMEM65 | PTPRA:copy21 | XIRP11 |
| CREB3 | linc-AKAP3-4:antisense | linc-TMEM72-2:copy2:antisense | PTPRA:copy31 | Y_RNA:RF00019.288 |
| CSRNP31 | linc-AKR1E2-2 | linc-TMEM72-2:copy3:antisense | PTPRA1 | YEATS41 |
| CSRP11 | linc-ALDH6A1 | linc-TMEM72-2:copy5:antisense | PTPRT | YIPF7 |
| CYP2W11 | linc-ARF6-1:antisense | linc-TNFAIP3-1:copy3 | PTS | ZC4H2 |
| DCAF8 | linc-ARHGAP11B-3 | linc-VLDLR-2 | PUS7L1 | ZFP691 |
| DCLRE1A1 | linc-ARHGAP20-3 | linc-ZFHX4-2:antisense | RAET1K | ZFPM2 |
| DDT1 | linc-ATP8A1-1:antisense | linc-ZNF208 | RARS1 | ZFR2:copy2 |
| DEFB107A1 | linc-BCL6-5:antisense | linc-ZNF99-6:antisense | RBM12 | ZNF235 |
| DGCR2 | linc-BCL6-6:antisense | LIPJ1 | RBM8A1 | ZNF302 |
| DIAPH3-AS1:antisense | linc-BEND4:copy2 | LOC100128568:antisense | REL1 | ZNF37BP |
| DKFZp686K1684:antisense | linc-C13orf1-3:antisense | LOC100129636:copy4 | REXO4 | ZNF4181 |
| DLGAP5 | linc-C15orf41-3 | LOC100129636:copy41 | RIMS1:copy4 | ZNF438 |
| DNAJC241 | linc-C20orf196-2:antisense | LOC100132352 | RNASEK1 | ZNF512 |
| DOK6 | linc-C8orf47 | LOC100132815:antisense | RNF1221 | ZNF615 |
| DRD5 | linc-CCKAR-8:copy2:antisense | LOC1002881421 | RNF5:copy21 | ZNF619:antisense |
| DSG41 | linc-CD93-1 | LOC1005056791 | RNF5:copy31 | ZNF8311 |
| DST1 | linc-CD93-1:copy2 | LOC254099:antisense | RNF5:copy41 | ZSCAN25 |

**Supplemental table 3**: **Shared overexpressed genes with established clinical outcome correlations. Overall survival (OS), progression free survival (PFS), disease free survival (DFS), measurable residual disease (MRD), relapse free survival (RFS)**

| Gene | Expression level | Clinical measure | Gene source | Cancer type | Ref |
| --- | --- | --- | --- | --- | --- |
| *AGPAT5* | High | Prognosis-variable | External database | Multiple | ^1^ |
| *ANGEL2* | High | ↓ OS | External database | Cervical | ^2^ |
| *BACE2* | High | ↓ OS | External database | Multiple | ^3^ |
|  |  |  |  |  |  |
| *BMI1* | High | ↓ OS | External database | Multiple | ^4^ |
| *CALCA* | High | ↓ OS, PFS | External database | Lung | ^5^ |
| *DLGAP5* | High | ↓ OS, DFS | External database | Multiple | ^6^ |
| *EIF4A1* | High | Advanced stage | Internal samples | Gastric | ^7^ |
| *HBS1L* | High | ↓ OS | External database | Ovarian | ^8^ |
| *KCNV2* | High | ↓ OS | External database | Breast | ^9^ |
| *LINC00210* | High | ↓ OS | Internal samples | Non-small cell lung carcinoma | ^10^ |
| *MANSC1* | Low | ↓ OS | External database | Acute myeloid leukemia | ^11^ |
| *MFSD2B* | High | ↓ OS | External database | Lung | ^12^ |
| *MRPS14* | High | ↓ OS | External database | Ovarian | ^13^ |
| *NHLRC3* | High | ↓ OS | Internal samples, external database | Colorectal carcinoma | ^14^ |
| *OCSTAMP* | High | ↑ OS, MRD | Internal samples | Multiple myeloma | ^15^ |
| *POMGNT1* | High | ↓ OS | Internal samples, external database | Glioblastoma Multiforme | ^16^ |
| *PTCSC3* | Low | ↓ OS | Internal samples | Gastric | ^17^ |
| *RAET1K* | High | ↓ OS | External database | Lung | ^18^ |
| *RBM12* | High | ↓ OS, PFS, RFS | Internal samples, external database | Liver | ^19^ |
| *REXO4* | High | ↓ OS | Internal samples, external database | Hepatocellular carcinoma | ^20^ |
| *SELENOO* | Low | ↓ OS | External database | Thyroid | ^21^ |
| *SF3B1* | Mutation | Low rate of metastasis | Internal samples | Uveal melanoma | ^22^ |
| *SHF* | High | ↑ OS | Internal samples | Neuroblastoma | ^23^ |
| *SIX6* | High | ↓ OS, ↓ RFS | External database | NSCLC, lung adenocarcinoma | ^24^ |
| *SLC41A3* | Variable | ↑ OS | External database | Multiple | ^25^ |
| *SNHG1* | High | ↓ OS | Internal samples | Gastric | ^26^ |
| *SPAG1* | Low | ↑ RFS | External database | Breast | ^27^ |
| *SYNDIG1* | High (8-gene signature) | ↓ RFS | External database | Breast | ^28^ |
| *TATDN2* | High (9-gene signature) | ↓ RFS | External database | Hepatocellular carcinoma | ^29^ |
| *ZNF691* | Low | ↑ OS | External database | Ovarian | ^30^ |

1. Fernández LP, Gómez de Cedrón M, Ramírez de Molina A. Alterations of Lipid Metabolism in Cancer: Implications in Prognosis and Treatment. *Front Oncol*. 2020;10:577420. doi:10.3389/fonc.2020.577420

2. Han Y, Ji L, Guan Y, et al. An epigenomic landscape of cervical intraepithelial neoplasia and cervical cancer using single-base resolution methylome and hydroxymethylome. *Clin Transl Med*. Jul 2021;11(7):e498. doi:10.1002/ctm2.498

3. Farris F, Matafora V, Bachi A. The emerging role of β-secretases in cancer. *J Exp Clin Cancer Res*. Apr 29 2021;40(1):147. doi:10.1186/s13046-021-01953-3

4. Wang MC, Li CL, Cui J, et al. BMI-1, a promising therapeutic target for human cancer. *Oncol Lett*. Aug 2015;10(2):583-588. doi:10.3892/ol.2015.3361

5. Zheng Q, Min S, Zhou Q. Identification of potential diagnostic and prognostic biomarkers for LUAD based on TCGA and GEO databases. *Biosci Rep*. Jun 25 2021;41(6)doi:10.1042/bsr20204370

6. Tang N, Dou X, You X, Shi Q, Ke M, Liu G. Pan-cancer analysis of the oncogenic role of discs large homolog associated protein 5 (DLGAP5) in human tumors. *Cancer Cell Int*. Aug 28 2021;21(1):457. doi:10.1186/s12935-021-02155-9

7. Gao C, Guo X, Xue A, Ruan Y, Wang H, Gao X. High intratumoral expression of eIF4A1 promotes epithelial-to-mesenchymal transition and predicts unfavorable prognosis in gastric cancer. *Acta Biochim Biophys Sin (Shanghai)*. Mar 18 2020;52(3):310-319. doi:10.1093/abbs/gmz168

8. Wang J, Xu J, Li K, et al. Identification of WTAP-related genes by weighted gene co-expression network analysis in ovarian cancer. *Journal of Ovarian Research*. 2020/09/30 2020;13(1):119. doi:10.1186/s13048-020-00710-y

9. Leung YK, Govindarajah V, Cheong A, et al. Gestational high-fat diet and bisphenol A exposure heightens mammary cancer risk. *Endocr Relat Cancer*. Jul 2017;24(7):365-378. doi:10.1530/erc-17-0006

10. Liu Z, Xu L, Zhang K, Guo B, Cui Z, Gao N. LINC00210 plays oncogenic roles in non-small cell lung cancer by sponging microRNA-328-5p. *Exp Ther Med*. May 2020;19(5):3325-3331. doi:10.3892/etm.2020.8593

11. Cheng Y, Su Y, Wang S, et al. Identification of circRNA-lncRNA-miRNA-mRNA Competitive Endogenous RNA Network as Novel Prognostic Markers for Acute Myeloid Leukemia. *Genes (Basel)*. Jul 31 2020;11(8)doi:10.3390/genes11080868

12. Bao L, Zhang Y, Wang J, et al. Variations of chromosome 2 gene expressions among patients with lung cancer or non-cancer. *Cell Biol Toxicol*. Oct 2016;32(5):419-35. doi:10.1007/s10565-016-9343-z

13. Sotgia F, Lisanti MP. Mitochondrial mRNA transcripts predict overall survival, tumor recurrence and progression in serous ovarian cancer: Companion diagnostics for cancer therapy. *Oncotarget*. Sep 15 2017;8(40):66925-66939. doi:10.18632/oncotarget.19963

14. Chen H, Sun X, Ge W, Qian Y, Bai R, Zheng S. A seven-gene signature predicts overall survival of patients with colorectal cancer. *Oncotarget*. Nov 10 2017;8(56):95054-95065. doi:10.18632/oncotarget.10982

15. Wang ZL, Liu Y, Zhou YL, et al. Osteoclast stimulatory transmembrane protein (OC-STAMP) is a promising molecular prognostic indicator for multiple myeloma. *Eur J Haematol*. Aug 2020;105(2):185-195. doi:10.1111/ejh.13425

16. Lan J, Guo P, Lin Y, et al. Role of glycosyltransferase PomGnT1 in glioblastoma progression. *Neuro Oncol*. Feb 2015;17(2):211-22. doi:10.1093/neuonc/nou151

17. Zhang G, Chi N, Lu Q, Zhu D, Zhuang Y. LncRNA PTCSC3 Is a Biomarker for the Treatment and Prognosis of Gastric Cancer. *Cancer Biother Radiopharm*. Feb 2020;35(1):77-81. doi:10.1089/cbr.2019.2991

18. Sui J, Yang S, Liu T, et al. Molecular characterization of lung adenocarcinoma: A potential four-long noncoding RNA prognostic signature. *J Cell Biochem*. Jan 2019;120(1):705-714. doi:10.1002/jcb.27428

19. Gao C, Shen J, Chen W, et al. Increased RBM12 expression predicts poor prognosis in hepatocellular carcinoma based on bioinformatics. *J Gastrointest Oncol*. Aug 2021;12(4):1905-1926. doi:10.21037/jgo-21-390

20. Ruan Y, Chen W, Gao C, et al. REXO4 acts as a biomarker and promotes hepatocellular carcinoma progression. *J Gastrointest Oncol*. Dec 2021;12(6):3093-3106. doi:10.21037/jgo-21-819

21. Zhao Y, Chen P, Lv HJ, et al. Comprehensive Analysis of Expression and Prognostic Value of Selenoprotein Genes in Thyroid Cancer. *Genet Test Mol Biomarkers*. Apr 2022;26(4):159-173. doi:10.1089/gtmb.2021.0123

22. Harbour JW, Roberson ED, Anbunathan H, Onken MD, Worley LA, Bowcock AM. Recurrent mutations at codon 625 of the splicing factor SF3B1 in uveal melanoma. *Nat Genet*. Feb 2013;45(2):133-5. doi:10.1038/ng.2523

23. Takagi D, Tatsumi Y, Yokochi T, et al. Novel adaptor protein Shf interacts with ALK receptor and negatively regulates its downstream signals in neuroblastoma. *Cancer Sci*. May 2013;104(5):563-72. doi:10.1111/cas.12115

24. Liu Q, Li A, Tian Y, et al. The expression profile and clinic significance of the SIX family in non-small cell lung cancer. *J Hematol Oncol*. Nov 8 2016;9(1):119. doi:10.1186/s13045-016-0339-1

25. Liu J, Zhang S, Dai W, Xie C, Li JC. A Comprehensive Prognostic and Immune Analysis of SLC41A3 in Pan-Cancer. *Front Oncol*. 2020;10:586414. doi:10.3389/fonc.2020.586414

26. Zhang H, Lu W. LncRNA SNHG12 regulates gastric cancer progression by acting as a molecular sponge of miR‑320. *Mol Med Rep*. Feb 2018;17(2):2743-2749. doi:10.3892/mmr.2017.8143

27. Lin S, Lv Y, Zheng L, Mao G, Peng F. Expression and Prognosis of Sperm-Associated Antigen 1 in Human Breast Cancer. *Onco Targets Ther*. 2021;14:2689-2698. doi:10.2147/ott.S288484

28. Cui Q, Tang J, Zhang D, et al. A prognostic eight-gene expression signature for patients with breast cancer receiving adjuvant chemotherapy. *J Cell Biochem*. Nov 6 2019;doi:10.1002/jcb.29550

29. Yu B, Liang H, Ye Q, Wang Y. Establishment of a Genomic-Clinicopathologic Nomogram for Predicting Early Recurrence of Hepatocellular Carcinoma After R0 Resection. *J Gastrointest Surg*. Jan 2021;25(1):112-124. doi:10.1007/s11605-020-04554-1

30. Luo X, Xu JG, Wang Z, et al. Bioinformatics Identification of Key Genes for the Development and Prognosis of Lung Adenocarcinoma. *Inquiry*. Jan-Dec 2022;59:469580221096259. doi:10.1177/00469580221096259
